# Functional improvement after hip arthroscopy in cases of active paediatric hip joint tuberculosis: a retrospective comparative study vis-à-vis conservative management

**DOI:** 10.1007/s11832-015-0705-5

**Published:** 2015-11-16

**Authors:** Vivek Tiwari, Shah Alam Khan, Ashok Kumar, Rishiram Poudel, Venkatesan Sampath Kumar

**Affiliations:** Department of Orthopaedics, All India Institute of Medical Sciences, Ansari Nagar, New Delhi, 110029 India

**Keywords:** Tuberculosis, Hip arthroscopy, Hip joint, Painful hip, Modified Harris hip score

## Abstract

**Purpose:**

Tuberculosis of the hip joint is a significant cause of preventable disability, especially in children. The aim of our study was to evaluate the functional results of hip arthroscopy done in a cohort of patients with hip joint tuberculosis and to compare them with the outcome of conservatively managed cases.

**Methods:**

This was a retrospective cohort study in which we evaluated the records of 22 hip arthroscopies performed in known cases of tuberculosis of the hip joint in children less than 12 years of age. A note of the demographic and clinical parameters like age, duration of symptoms, stage of the disease, time period of follow-up, any complications during surgery, and pre- and post-operative modified Harris hip score (MHHS) was made in all cases. We compared the results with an age-matched cohort of 44 children with hip joint tuberculosis who were treated non-operatively with anti-tuberculosis therapy and traction in the same tertiary care institute.

**Results:**

The arthroscopic findings in our series included synovitis, chondral erosions of the femoral head and/or acetabulum, pannus formation over the femoral head and/or acetabulum, and labral tears. The various arthroscopic procedures which were done included joint lavage, synovectomy, labral debridement and cheilectomy. The mean follow-up was 45 months, with the minimum being 36 months. There was a statistically significant change in the mean MHHS after hip arthroscopic procedures (*p* < 0.001); the difference in the mean post- and pre-operative MHHS was independent of age, stage or duration of follow-up. There was a statistically significant difference (*p* < 0.05) between the magnitude of improvement in MHHS after hip arthroscopy and that after conservative management.

**Conclusions:**

Arthroscopy of the hip joint in children in cases of tuberculosis can serve as an emerging therapeutic modality. It is an effective and safe minimally invasive procedure, and helps in improving the functional outcome in early disease.

## Introduction

Tuberculosis of the hip joint in children is a common problem in the developing world, constituting 15 % of cases of all forms of osteo-articular tuberculosis [1]. Although there has been a gradual reduction in the incidence of osteo-articular tuberculosis, the complications and sequelae of tuberculosis of the hip joint in children continue to be a major problem, particularly in the third world. Treatment guidelines are well defined for pulmonary tuberculosis but the same cannot be said with certainty for extra-spinal musculoskeletal tuberculosis, more so for the paediatric population that forms more than half of the case-load. The majority of the studies on hip joint tuberculosis recommend conservative treatment of the condition, with emphasis on anti-tubercular therapy (ATT) and traction [2, 3]. Surgical intervention is done in few cases and is limited to synovectomy, cold abscess drainage and debridement of the hip joint [4]. As surgical intervention carries the risk of increasing the morbidities and worsening the condition, especially in the paediatric population, a need for less invasive procedures for treatment was felt. Arthroscopy of the hip in children has very limited indications and, to the best of our knowledge, there are no studies that describe the arthroscopic findings or procedures in tuberculosis of the hip joint in children, although it has been described in septic arthritis [5]. Besides providing a minimally invasive option for surgical management, arthroscopy in hip joint tuberculosis in children can be helpful in knowing the exact geographical extent of the disease. Arthroscopic procedures like arthroscopic synovectomy and debridement carry the advantage of being minimally invasive and offer an alternative to more radical open hip procedures in the management of tuberculosis of the hip joint in the growing skeleton.

The aim of the present study was to evaluate the arthroscopic findings and functional outcome in patients with tuberculosis of the hip joint undergoing arthroscopic procedures and to compare the clinical outcomes of the arthroscopic procedures among different clinico-radiological stages of the disease. We also compared the functional outcome after arthroscopic procedures with that of a cohort of age-matched conservatively managed cases of hip joint tuberculosis and to suggest an arthroscopy-based classification for hip joint tuberculosis in children.

## Materials and methods

This was a retrospective cohort study done between January 2008 and February 2013. An operative group consisting of consecutive cases of tuberculosis of the hip joint in children, who underwent hip arthroscopy, was retrospectively evaluated after obtaining clearance from our Hospital Ethics Committee (reference no. IEC/NP-381/2013, date 08/08/2013). A control group of cases with paediatric hip joint tuberculosis who were conservatively managed with traction and ATT was also retrospectively evaluated. Children less than 12 years of age (as it is the cut-off limit for paediatric registration in our institute) with a known diagnosis of tuberculosis of the hip joint and who had taken ATT from our institute were included in the study. Children who took ATT from outside our institute were not included in order to avoid treatment bias. The diagnosis of tuberculosis was made on the basis of demonstration of *Mycobacterium tuberculosis* bacteria on culture of the synovial fluid aspirated from the hip. For the clinico-radiological grading, we had used the conventional classification, which divides joint tuberculosis into stage of synovitis, stage of early arthritis, stage of late arthritis and stage of destruction/sequelae [6] (Table 1). Children with stage 4 tuberculosis were excluded from the study. During the study period, 27 cases of paediatric hip joint tuberculosis underwent hip arthroscopy, of which five cases had taken ATT from outside our institute, which were subsequently removed from the study. Therefore, the operative group comprised 22 cases which fulfilled all the inclusion criteria. We came across 110 cases of paediatric hip joint tuberculosis which were conservatively managed in the study period. Of the above cases, 33 patients had taken ATT from outside the institute, records were incomplete for 16 cases and 12 patients were in clinico-radiological stage 4 of hip joint tuberculosis. Age-matching was done for the remaining 49 cases and a control group of 44 cases was recruited. The two study groups were separate and there were no cross-overs. Data were retrieved from hospital records and recorded on a pre-fixed proforma. A note of the demographic and clinical parameters like age, gender, duration of symptoms, period of follow-up and the clinico-radiological stage of tuberculosis was made. All patients were graded by a single observer. All children had taken ATT consisting of four drugs. Since drug resistance to ATT is very high in this part of the world, the standard protocol in bone and joint tuberculosis at our hospital involves using four drugs (rifampicin, isoniazid, pyrazinamide and ethambutol) for 4 months, three drugs (rifampicin, isoniazid and ethambutol) for the subsequent 3 months and two drugs (rifampicin, isoniazid) for a further 11 months. We did not test for drug resistance in our study population.

**Table 1:** Clinico-radiological grading of tuberculosis of the hip

Stage	Features	Deformity	Limb length
Stage 1	Stage of synovitis	Flexion, abduction, external rotation	Apparent lengthening
Stage 2	Stage of early arthritis	Flexion, adduction, internal rotation	Apparent shortening
Stage 3	Stage of late arthritis	Flexion, adduction, internal rotation	True shortening
Stage 4	Stage of destruction	Flexion, adduction, internal rotation	Gross true shortening

All children in the operative group underwent hip arthroscopy in the supine position with the hip joint distracted on a fracture table, guided by an image intensifier. In our study, indications for arthroscopy were persistent pain in a case of tuberculosis of the hip not relieved by traction and ATT for 2 months. All hip arthroscopies were done by the senior author (SAK). We used the anterior, anterolateral and anteromedial portals for accessing the hip joint. A note of the arthroscopic findings was made in all cases in the form of written operation notes and pictures. All children had received a minimum of 2 months of ATT before undergoing hip arthroscopy. Post-operatively, patients were advised bed rest for 2 days followed by partial weight-bearing mobilisation and physiotherapy in the form of hip range-of-motion exercises.

Children in the control group were given skin traction for 2 months along with ATT. After 2 months, hip range-of-motion exercises were started, along with partial weight-bearing mobilisation.

In both groups, the functional activity level was evaluated using a modified Harris hip score (MHHS) (Table 2). In the operative group, the MHHS was first evaluated just before performing the hip arthroscopy. The post-operative MHHS was evaluated at the final follow-up. In the control group, the MHHS was evaluated at the time of diagnosis and also at the final follow-up.

**Table 2:** Modified Harris hip score (MHHS)

Points	Description
*Pain*	
44	None/ignores
40	Slight, occasional, no compromise in activity
30	Mild, no effect on ordinary activity, pain after activity, uses aspirin
20	Moderate, tolerable, makes concessions, occasional codeine
10	Marked, serious limitations
0	Totally disabled
*Function*	
Limp	
11	None
8	Slight
5	Moderate
0	Severe
0	Unable to walk
Support	
11	None
7	Cane, long walks
5	Cane, full time
4	Crutch
2	2 canes
0	2 crutches
0	Unable to walk
Distance walked	
11	Unlimited
8	6 blocks
5	2–3 blocks
2	Indoors only
2	Bed and chair
*Functional activities*	
Stairs	
4	Normally
2	Normally with banister
1	Any method
0	Not able
Socks/shoes	
4	With ease
2	With difficulty
0	Unable
Sitting	
5	Any chair, 1 h
3	High chair, 0.5 h
0	Unable to sit, 0.5 h, any chair
Public transportation	
1	Able to enter public transportation
0	Unable to use public transportation

The Harris hip score includes 91 points for pain and function and 9 points for range of motion and deformity. The range of motion and deformity portion has been omitted. The multiplier 1.1 is used to give a total possible score of 100: total score = total points × 1.1

Statistical analysis was done using SPSS 20.0 software (IBM, Inc.). The Shapiro–Wilk test was used to test the normal distribution of data. A paired *t*-test was applied to find out the significance of differences in the pre- and post-operative MHHS in the operative group and the pre- and post-treatment MHHS in the control group. A Kruskal–Wallis analysis of variance (ANOVA) test was applied to find whether age, stage or follow-up duration of the patients affected the difference in the MHHS in both groups. Student’s *t*-test was applied to evaluate whether there was any statistically significant difference in the improvement of the MHHS when compared between the operative and control groups.

## Results

**Fig. 1:**
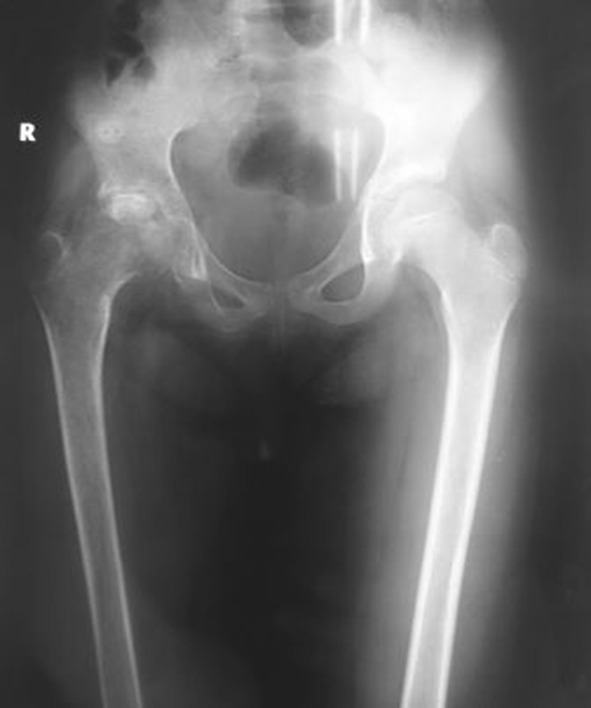
Plain radiograph of the pelvis with both hips of a 10-year-old with right side hip joint tuberculosis (clinico-radiological stage 2), which was treated with arthroscopic debridement

There were 14 males and eight females in the operative group. The mean age of the patients was 10.18 years (with a range of 6–12 years). The mean follow-up was 45 months, with the minimum being 36 months. Seventeen children presented with pain in the hip region, while five children presented with knee pain. The average duration of symptoms in the group, prior to arthroscopy, was 3 months (with a range of 2–6 months). Of these, 11 patients were in stage 1, seven patients were in stage 2 and four patients were in stage 3 of the disease process (Fig. 1).

**Fig. 2:**
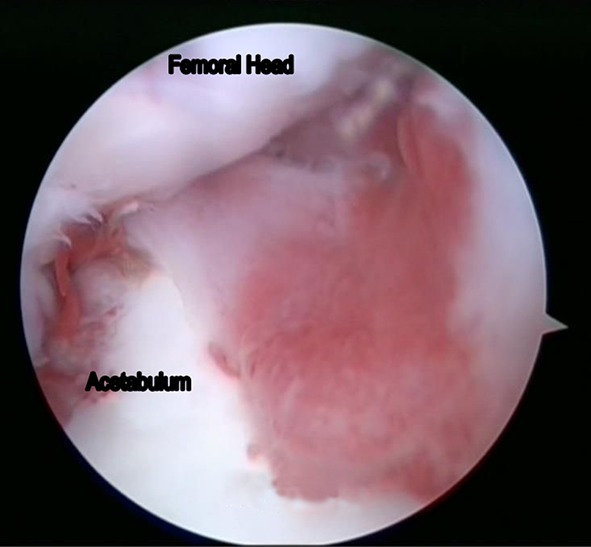
Arthroscopic picture showing pannus formation over the acetabular fossa in tuberculosis of the hip joint in an 11-year-old child (arthroscopic stage I)

**Fig. 3:**
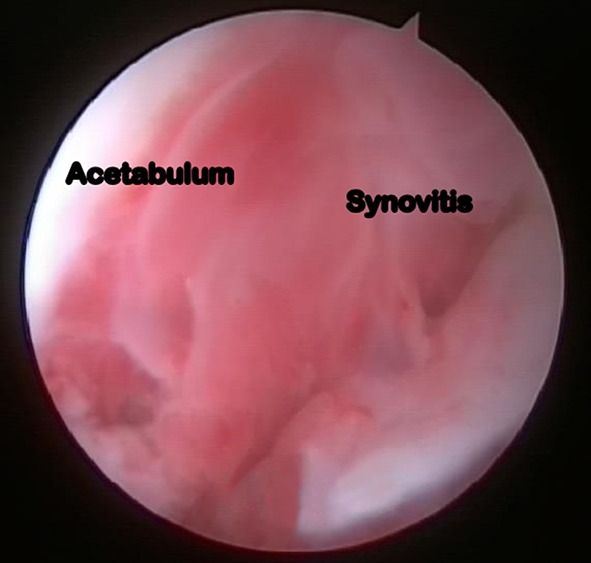
Arthroscopic image depicting intense synovitis in an 11-year-old with tuberculosis of the hip joint (arthroscopic stage I)

**Fig. 4:**
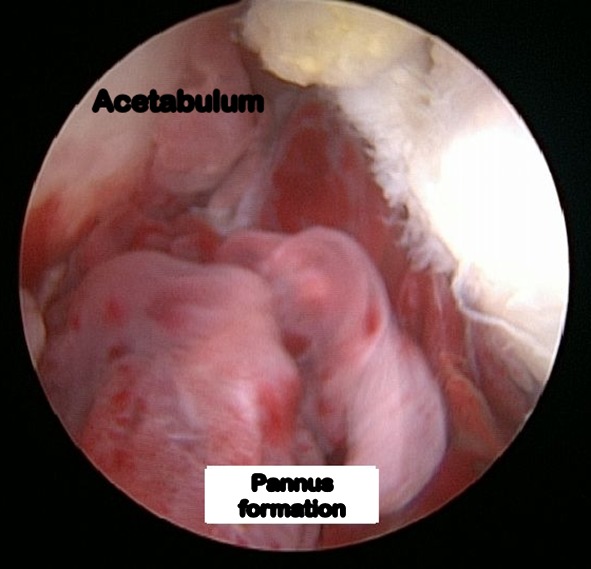
Arthroscopic picture showing hyperaemia and pannus formation on the floor of the acetabulum with non-visualisation of the labrum in a 10-year-old with tuberculosis of the hip joint (arthroscopic stage II)

**Fig. 5:**
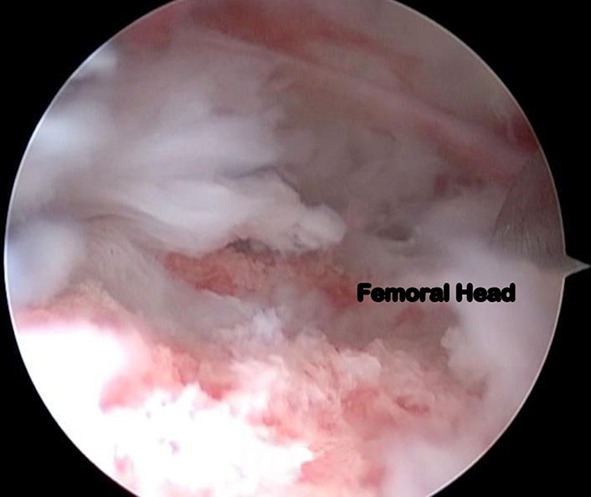
Another arthroscopic photograph depicting extensive destruction with cartilage erosion in tuberculosis of the hip joint in an 11-year-old boy (arthroscopic stage III)

Debridement of the hip joint combined with synovectomy was the most common procedure carried out in our series (*n* = 10). This was followed by synovectomy (*n* = 6) and debridement (*n* = 5). We performed partial cheilectomy in one patient. The arthroscopic findings in our series included synovitis, chondral erosions of the femoral head and/or the acetabulum, pannus formation over the femoral head and/or the acetabulum, and labral tears (Figs. 2, 3, 4 and 5). None of the hips in our series had a cold abscess. This was probably because all children in the study had received a minimum of 2 months of ATT before undergoing hip arthroscopy. No complications were noted in any patient post-arthroscopy. None of the hips dislocated. There was no loss to follow-up.

The mean pre- and post-operative MHHS were 58.64 and 76.82, respectively, with mean difference of 18.18. Difference in the overall post- and pre-operative scores was found to be highly significant (*p* < 0.001). The mean values of the MHHS in the different clinico-radiological stages are described in Table 3. It was seen that the difference in the MHHS was significant in each stage of tuberculosis of the hip independently (*p* < 0.001). A complete profile of all the cases with pre- and post-arthroscopy MHHS is shown in Table 4. The difference in the mean post- and pre-operative MHHS was found to be independent of age, stage or duration of follow-up of the patients (*p* < 0.001).

**Table 3:** Mean MHHS according to the different clinico-pathological grades of the disease

Stage of the disease	Operative/control group	Mean pre-operative/pre-treatment MHHS	Mean post-operative/post-treatment MHHS	Mean difference in MHHS	*p*-Value
Stage 1	Operative	65.91	84.10	18.18	<0.001
	Control	64.75	74.50	9.75	<0.001
Stage 2	Operative	55.71	74.29	18.57	<0.001
	Control	50.58	66.17	15.59	<0.001
Stage 3	Operative	43.75	61.25	17.5	<0.001
	Control	45.0	57.14	12.14	<0.001

**Table 4:** Demographic profile of patients in the operative group along with outcome data

S. no.	Age (years)/gender	Pre-operative stage (clinico-radiological classification)	Pre-operative MHHS	Post-operative MHHS	Difference between pre- and post-operative MHHS	Follow-up period (months)	Arthroscopic stage
1	11/M	1	55	85	30	41	I
2	11/M	1	60	75	15	43	I
3	12/M	2	55	70	15	47	I
4	12/M	1	70	85	15	46	II
5	8/M	2	55	80	25	44	I
6	9/M	2	50	75	25	46	II
7	11/F	1	65	90	25	52	I
8	12/F	1	70	75	5	57	II
9	7/M	1	65	90	25	42	I
10	6/M	2	45	65	20	40	II
11	9/F	3	40	55	15	43	II
12	11/F	1	65	90	25	43	II
13	11/M	3	40	60	20	44	III
14	12/F	1	70	85	15	43	II
15	8/M	1	65	80	15	50	I
16	10/M	2	60	80	20	49	II
17	12/M	3	50	55	5	42	II
18	12/M	1	70	90	20	43	I
19	11/M	2	65	85	20	58	I
20	10/F	1	70	80	10	44	II
21	10/F	2	60	65	5	42	III
22	9/F	3	45	75	30	36	I

There were 30 male and 14 female patients in the control group. The number of patients in stages one, two and three were 20, 17 and 7, respectively. The mean follow-up was 47 months, with the minimum being 36 months. The mean pre- and post-treatment MHHS were 58.18 and 68.52, respectively, with a mean difference of 10.34. The improvement in the score was found to be statistically significant (*p* value <0.05). The disease stage, age and duration of follow-up of the patients was not found to affect the quantum of improvement (*p* value <0.05). The mean values of the MHHS in the different clinico-radiological stages are described in Table 3. It was seen that the difference in the MHHS was significant in each stage of tuberculosis of the hip independently (*p* < 0.001). A complete profile of all the cases with pre- and post-treatment MHHS is shown in Table 5.

**Table 5:** Demographic profile of patients in the control group along with outcome data

S. no.	Age (years)/gender	Stage (clinico-radiological classification)	Pre-treatment MHHS	Post-treatment MHHS	Difference between pre- and post-treatment MHHS	Follow-up period (months)
1	12/F	1	60	70	10	40
2	10/M	2	55	65	10	50
3	10/M	1	65	70	5	53
4	7/F	1	60	65	5	52
5	6/M	3	45	55	10	51
6	6/M	2	50	60	10	37
7	8/M	3	40	50	10	38
8	12/F	1	65	75	10	39
9	11/M	2	55	70	15	36
10	11/F	2	60	75	15	43
11	9/F	1	65	70	5	48
12	8/M	2	50	60	10	54
13	11/M	1	65	80	15	48
14	12/F	1	65	70	5	58
15	8/M	1	60	80	20	59
16	11/M	1	70	75	5	49
17	12/M	2	55	65	10	55
18	12/M	1	60	75	15	43
19	8/F	2	60	65	5	36
20	9/M	1	70	80	10	42
21	10/M	3	50	60	10	48
22	7/M	1	65	75	10	57
23	11/M	2	55	65	10	44
24	10/F	2	60	75	15	47
25	12/M	1	65	80	15	49
26	11/M	1	65	70	5	58
27	9/F	2	60	65	5	54
28	11/M	1	70	80	10	41
29	9/M	3	45	50	5	40
30	12/M	3	40	60	20	54
31	10/M	2	65	70	5	44
32	11/M	1	65	70	5	46
33	9/M	1	65	75	10	50
34	12/F	2	55	65	10	47
35	11/M	2	50	60	10	44
36	12/M	3	45	60	15	41
37	9/M	1	65	70	5	48
38	12/F	2	55	65	10	44
39	11/F	2	60	70	10	42
40	10/M	2	50	60	10	38
41	11/F	1	65	80	15	37
42	12/M	3	50	65	15	45
43	12/M	2	55	70	15	48
44	11/F	1	65	80	15	55

There was a statistically significant difference (*p* < 0.05) between the magnitude of improvement in the MHHS after hip arthroscopy and that after conservative management.

### Arthroscopic grading of tuberculosis of the hip

In the operative group, we found that patients in whom the labrum appeared to be damaged had a worse functional outcome (as seen by the MHHS) as compared to those in whom the labrum was intact. Based on these findings, we devised a grading system for hip joint tuberculosis, as follows:

- Grade I: Inflammatory changes seen in the periphery of the joint and/or inflammatory changes in the acetabular fossa. All anatomic hip structures are intact. It included the presence of mild/partial articular cartilage damage.
- Grade II: Presence of labral tear with/without grade I changes.
- Grade III: Total destruction of the hip with no visible integrity of the internal structures.

In our study, the number of patients in grades one, two and three were 9, 11 and 2, respectively.

The median values of the MHHS in different arthroscopic grades are described in Table 6. There was a regular decline in the improvement of the MHHS after hip arthroscopy as one goes down the arthroscopic grades, validating our classification, although the difference in the improvement of the MHHS as per arthroscopic grades was not found to be statistically significant (*p* = 0.08). As the quantum of difference appeared to be large, statistical insignificance can be explained by the small sample size in the study. We calculated Pearson’s correlation coefficient to correlate the three grades of arthroscopic classification with the corresponding stages of clinico-radiologic classification. It was found to be 0.32, signifying a weak relationship. Moreover, the mean improvement in functional outcome after arthroscopy, as measured by the MHHS, was better in clinico-radiologic stage 2 patients as compared to stages 1 and 3 patients. Since our functional classification was validated by a progressive decrease in the median quantum of improvement in the MHHS as one goes down from grades one to three, the findings signify pondering over the validity of the clinico-radiologic classification.

**Table 6:** Median MHHS according to the different arthroscopic grades of the disease

Grade of the disease	Median pre-operative MHHS	Median post-operative MHHS	Median difference in MHHS	*p*-Value
Grade 1	62.5	82.5	20	<0.001
Grade 2	62.5	77.5	15	<0.001
Grade 3	50	62.5	12.5	<0.001

## Discussion

Tuberculosis has been one of the most intriguing problems for the medical fraternity in the developing world. In tubercular infections of the bones and joints, the hip joint ranks second only to tuberculosis of the spine [7]. The introduction of chemotherapy revolutionised the medical management of tuberculosis by reducing the duration of the disease and decreasing the relapse rate [8]. However, in tuberculosis of the hip joint, the final outcome is poor despite chemotherapy, as there is widespread damage to vital intra-articular structures, leading to deformities and fibrous ankylosis. Surgical management comes to play an important role in the form of debridement and wash for the longevity of the normal function of the joint [6]. However, children often present late in the disease process, with delay in diagnosis. Transient synovitis and Legg–Calvé–Perthes disease are the two main differentials in such patients that need to be ruled out [9]. Juxta-articular osteoporosis, peripherally located osseous erosions and gradually decreasing joint space (referred to as Phemister’s triad) are some radiological signs which help in the early diagnosis on plain radiographs [10]. Delay in accurate diagnosis leads to advanced changes in the joint which cannot be managed with chemotherapy alone [1]. On the other hand, open surgical procedures carry with them the risk of aggravating the disease besides the peri-operative morbidities. Therefore, in such circumstances, requirement of other less invasive modalities of treatment become imperative. Moreover, there is a need for better evaluation tools, which could diagnose and treat as well as prognosticate the tubercular infection of the hip joint early.

Untreated tuberculosis of the hip has been described to pass through four stages based on the findings of plain radiographs: stage of synovitis, stage of early arthritis, stage of late arthritis and stage of destruction [6]. Joint debridement and synovectomy has been described as a treatment modality for stage III and stage IV patients [6]. But the objective improvement in such patients after the procedure has not been reported in the literature. Moreover, invasive surgery in these patients can predispose to intra-articular adhesions and fibrosis. We, thus, felt that, to diagnose and prognosticate the disease process and to prevent invasive surgery in these cases, hip arthroscopy could be safely used.

Hip arthroscopy is a technically demanding procedure and has a steep learning curve [11]. With the development of longer instruments, the new era of hip arthroscopy started in the early 1980s [12]. The role of hip arthroscopy in children was elucidated much later. The first account of hip arthroscopy in children came from Gross in 1977 [13]. Some of the present indications for hip arthroscopy in paediatric patients include Legg–Calvé–Perthes disease, labral tears, slipped capital femoral epiphysis and septic arthritis [14]. However, there are no reports of hip arthroscopy in tuberculosis of the hip joint. Hip arthroscopy confers certain advantages over conventional arthrotomy, including earlier return to activity, less invasive nature and less peri-operative morbidity. Moreover, there is no need for dislocation of the femoral head [15]. It, thus, avoids any potential damage to the vascularity of the femoral head. However, hip arthroscopy also carries certain risks. Complications of hip arthroscopy include sciatic and femoral nerve neurapraxia, fluid extravasation, transient pudendal nerve palsy, instrument breakage, focal myositis ossificans and triradiate cartilage damage [16, 17].

Healing of tuberculosis of the hip joint has been described with traction and ATT in 98 % of the cases [18]. Moreover, surgically intervening early during active disease has a risk of flare-up of the disease. Therefore, we resorted to operative management only after 2 months of ATT to reduce the chances of flare-ups and to see if conservative management was benefitting the patient.

In concurrence with recent recommendations for functionally evaluating hip arthroscopy patients, we used the MHHS to assess the functional outcome of our patients before and after hip arthroscopy [19].

We found that the mean improvement in the MHHS after hip arthroscopy in all the patients considered together was 18.18, which was found to be statistically significant (*p* < 0.001). However, the improvement varied in amount among different patients.

We found that involvement of the labrum in the hip joint in tuberculosis gives poor functional outcome. The presence of labral tear can, thus, serve as a watershed for treatment decisions in such patients. The labrum helps in deepening the acetabulum, thereby decreasing stresses on the hip joint and increasing its stability [20, 21]. Labral tears may affect the stability of the hip joint [22]. Labral tears are also believed to result in premature articular damage, which may further affect the functional characteristics and mobility of the hip joint [23, 24]. Affection of the acetabular labrum (arthroscopic grade 2) in tuberculosis of the hip thus points to a poor prognosis, validating our arthroscopic classification of tuberculosis of the hip joint.

Our study has its own limitations. Small sample size and a non-randomised study design are our main drawbacks. Moreover, whether the natural process of the disease passes from the arthroscopic grade 1 towards arthroscopic grade 3 is not known. Also, whether hip arthroscopy alters the natural course of the disease process is not known. Besides, we did not have magnetic resonance imaging (MRI) performed for children in the conservatively managed group, as they had a good functional outcome with traction and ATT and, at that time, in the absence of symptoms, it was not required.

Arthroscopy of the hip joint in children in cases of tuberculosis can serve as an emerging therapeutic modality providing rapid and significant relief to the patients, besides confirming the diagnosis by providing tissue samples for histopathology and culture. It is a safe and minimally invasive procedure, improves the functional outcome with less operative morbidity and can be done as a day care surgery. Tuberculosis of the hip with involvement of the labrum has a poor prognosis vis-a-vis functional outcome. Future prospective long-term follow-up studies are recommended on the subject.
